# Exploring the Bacterial Communities of the Kaiafas Thermal Spring Anigrides Nymphes in Greece Prior to Rehabilitation Actions

**DOI:** 10.3390/ijerph17239133

**Published:** 2020-12-07

**Authors:** Agapi I. Doulgeraki, Vasiliki Bikouli, Anthoula A. Argyri, Nikos Chorianopoulos, Elisavet Mitre, Georgia Charvourou, Patra Sourri, Chrysoula C. Tassou, Alexandra Oikonomou

**Affiliations:** 1 Institute of Technology of Agricultural Products, Hellenic Agricultural Organization-DIMITRA, Sof. Venizelou 1, 14123 Lycovrissi, Greece; mpvicky@otenet.gr (V.B.); anthi.argyri@gmail.com (A.A.A.); nchorian@nagref.gr (N.C.); polyelicd@gmail.com (E.M.); gcharvourou@gmail.com (G.C.); patrapsourri@gmail.com (P.S.); ctassou@nagref.gr (C.C.T.); 2 Ephorate of Palaeoanthropology and Speleology, Hellenic Republic Ministry of Culture and Sports, Ardittou 34b, 11636 Athens, Greece; aloikonomou@culture.gr

**Keywords:** Kaiafas, thermal spring, water microbiome, metataxonomics, biodiversity

## Abstract

Anigrides Nymphes of Lake Kaiafas is a thermal spring that is well known for its therapeutical properties, as the hot water (32–34 °C) is rich in sulfur compounds and minerals. Nowadays, efforts are made from the Hellenic Republic to modernize the existing facilities and infrastructure networks of the area. To study the complex ecosystem of the thermal spring, we collected water from four sampling points (Lake, and Caves 1, 2, and 3). Filtration method was used for microbial enumeration. In parallel, total bacterial DNA was extracted and subjected to next-generation sequencing (NGS). A total of 166 different bacterial families were detected. Differences in families, genera, and species abundances were detected between the different sampling points. Specifically, *Comamonadaceae* was the most common family detected in Lake and Cave 3. Similarly, in Caves 1 and 2, *Rhodobacteraceae* was detected at a higher percentage compared to the rest of the families. Moreover, the detection of sequences assigned to waterborne or opportunistic pathogens, i.e., *Enterobacteriaceae*, *Legionellaceae*, *Coxiellaceae*, and *Clostridiaceae*, as well as *Enterococcus* and *Vibrio*, is of great importance. Although the presence of pathogens was not examined by quantitative PCR, the detection of their sequences strengthens the need of the planned rehabilitation actions of this natural environment in order to allow human swimming.

## 1. Introduction

Thermal springs all over the world attract the attention of citizens or travelers given the health-giving properties of their waters. It is well known from ancient times that swimming or even drinking water rich in minerals can comfort the healing of a variety of illnesses and promote health and wellness [1]. Therapists believe that bathing in thermal springs could treat heart diseases, musculoskeletal disorders, respiratory inflammation, and gynecological disorders including infertility [1,2,3]. However, the beneficial and therapeutical effect of thermal spring water on human health presupposes the ensured microbiological quality of waters. The presence of *Escherichia coli*, *Shigella*, *Legionella, Pseudomonas, Mycobacterium, Staphylococcus aureus*, and *Leptospira* in waters could constitute microbial potential hazards [4]. Swimming in microbially contaminated waters has been associated with several illnesses including legionellosis and folliculitis [4,5,6]. However, it is difficult to control contamination of water by fecal or waterborne microorganisms and subsequently manage the disinfection of the waters, especially in the case of natural spas, considering the natural properties including mineral components and natural microbiota should be respected [7].

In Greece, one of the most famous thermal springs from antiquity is Kaifas. Lake Kaiafas is a natural water ecosystem located in regional unit of Ileia of Western Greece Prefecture in of Greece, which is protected by the nature protection area network of the European Union—Natura 2000 (GR2330005). The water’s temperature is 32–34 °C, and high concentrations of sulfur compounds and minerals have been detected [8,9]. The thermal spring was found from antiquity to have therapeutical properties suitable for rheumatic, arthritic, and skin pathisis [8]. For many years, the area of Lake Kaifa was closed to the public by the Hellenic Republic. Nowadays, efforts are being made to modernize the existing facilities and infrastructure networks of the area, including rehabilitation actions. One of the main goals of these efforts is to better understand the risk of swimming in this area from a microbiological point of view in terms of designing a more effective disinfection management in the future. In this research, a deep study of the microorganisms present in water gushed out from the spring of Anigrides Nymphes was performed by microbiological and metagenomics analysis of water. In parallel, estimation of contamination sources was attempted by considering the presence of specific microorganisms associated with environmental contamination. The impact of swimming in the water in terms of the current situation on human health is also discussed on the basis of the metataxonomic analysis; however, the presence of pathogenic bacteria was not examined by quantitative PCR in this study.

## 2. Materials and Methods

### 2.1. Water Sampling

Water samples were collected from the Kaiafas thermal spring Anigrides Nymphes, located in the regional unit of Ileia of Western Greece Prefecture (Greece). Four sampling points were demarcated, including the “Lake” (swimming place) and the points of 3 caves (Caves 1, 2, and 3) (Figure S1). From each sampling point, 3 samples were obtained from 30 cm depth below the water’s surface [10]. The samples (1000 mL) were collected in a sterile water sampling bottle (PET, sterile without thiosulfate, Labbox), transferred under cooling conditions (<4 °C) in the laboratory, and analyzed within 24 h.

### 2.2. Microbiological Analysis

Filtration method was used for the analyses of the water samples [11]. For each microbiological analysis, a sample volume of 100mL was poured into a filter funnel and drawn through a membrane filter (0.45 μm) [12]. A sterile tweezer was used to transfer aseptically the membrane filter on the surface of each microbiological medium. Specifically, the total viable counts (TVC; grown on 22 °C and 37 °C) of *Enterococcus*, coliforms, *Escherichia coli*, *Pseudomonas aeruginosa*, and *Legionella* were enumerated according to European and Greek legislation [10,12]. The results were expressed as a mean value of colony forming units (CFU)/mL of the triplicates. In the case of *Legionella*, the mean value of CFU/mL represented the presumptive colonies detected on Buffered Charcoal Yeast Extract (BCYE) *Legionella* agar without performing biochemical tests for *Legionella* confirmation.

### 2.3. Sequencing Analysis

DNeasy PowerWater Kit (Qiagen, Hilden, Germany) (Company, City, Country)was used to extract the DNA from each sample. A volume of 100 mL from each sample (3 samples per sampling point) was filtrated and bulk DNA was prepared from each sampling point (Lake, and Caves 1, 2, and 3). After amplification of V2-4-8 and V3-7-9 hypervariable regions of 16S Ribosomal ribonucleic acid (rRNA) gene using an Ion 16S Metagenomics kit (Thermo Fisher Scientific, Waltham, MA, USA), sequencing of 400 bp amplicons was performed using Ion Torrent PGM by CeMIA SA (https://cemia.eu/) (Larissa, Greece). The resulting sequences were analyzed with Ion Reporter software (Thermo Fisher Scientific, Waltham, MA, USA). Bacterial operational taxonomic units (OTUs) were detected and taxonomically classified (at >97% similarity) according to Spyrelli et al. [13] using Nucleotide Basic Local Alignment Search Tool (BLASTn) against the National Center for Biotechnology Information (NCBI) database (www.ncbi.nlm.nih.gov) (Bethesda MD, 20894 USA).

### 2.4. Statistical Analysis

Significant difference among OTU abundance was considered by analysis of variance (ANOVA) at a 95% confidence level determined by Duncan’s multiple range test. The online platform MetaboAnalyst 4.0 (www.metaboanalyst.ca) was used to perform principal component analysis (PCA) on the 166 OTUs (bacterial families; X variables) detected in the thermal water samples (Lake, Cave 1, Cave 2, and Cave 3; Y variables) by metataxonomic analysis.

## 3. Results

Microbiological quality of water samples collected from four sampling points of the Kaiafas thermal spring Anigrides Nymphes was estimated by microbiological and metataxonomic analysis. In Table 1, the population of TVC of *E. coli*, coliforms, *Enteroccocus*, *Pseudomonas aeruginosa*, and *Legionella* are presented as a mean of the three samples collected from each sampling point. No significant differences were observed among the different samples on the measured microbiological parameters (*p* > 0.05).

**Figure 1:**
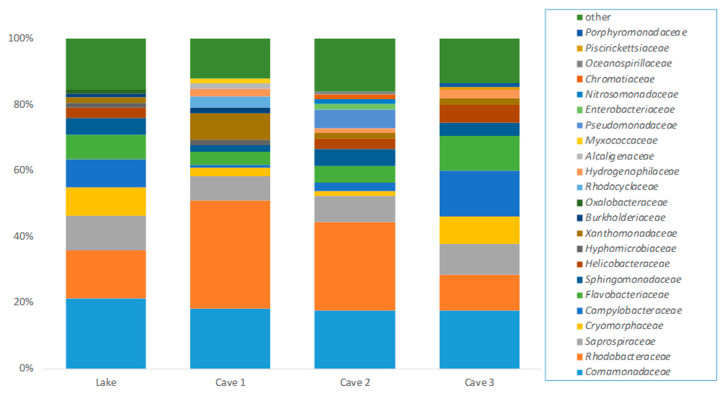
Abundance of families detected in thermal waters of Lake, Cave 1, Cave 2, and Cave 3 of the Kaiafas thermal spring Anigrides Nymphes in Greece. The term “other” represents the sum of the families with relative abundance <1%.

In total, 166 OTUs were detected in all samples, i.e., Lake (127 OTUs), Cave 1 (108 OTUs), Cave 2 (116 OTUs), and Cave 3 (116 OTUs). In Figure 1, the abundance of families (>1%) detected in thermal waters of the different sampling points are presented, whereas PCA mapping of the tested samples is shown in Figure S2. In brief, *Comamonadaceae* was the most common family detected in samples collected from Lake and Cave 3, followed by *Rhodobacteraceae* and *Campylobacteraceae*.

**Figure 2:**
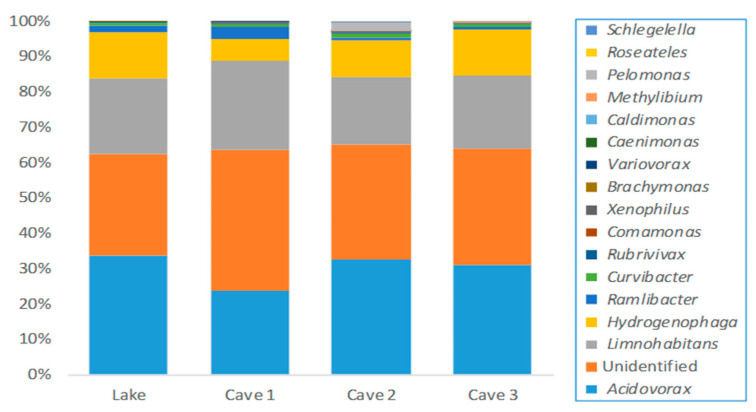
Relative abundance of the main genera of *Comamonadaceae* family detected in thermal waters of Lake, Cave 1, Cave 2, and Cave 3 of the Kaiafas thermal spring Anigrides Nymphes in Greece.

**Figure 3:**
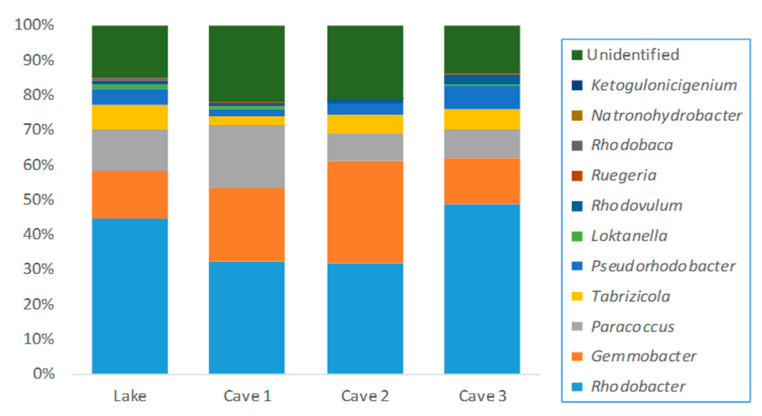
Relative abundance of the main genera of *Rhodobacteraceae* family detected in thermal waters of Lake, Cave 1, Cave 2, and Cave 3 of the Kaiafas thermal spring Anigrides Nymphes in Greece.

In terms of the samples collected from Cave 2 and Cave 3, the abundance of *Rhodobacteraceae* was higher than the rest of the families, followed by *Comamonadaceae*. The observed genera assigned to *Comamonadaceae* and *Rhodobacteraceae* are shown in Figure 2 and Figure 3, respectively, wherein *Acidorovax* and *Rhodobacter* were detected at a higher percentage than the rest of the genera, respectively. *Limnohabitans, Hydrogenophaga, Gemmobacter, Paracoccus, Tabriozicola,* and *Pseudorhodobacter* were also observed (>1%). *Xanthomonadaceae* was detected in high abundance (1.6 to 8.2 relative abundance) where the genera *Dyella, Xanthomonas, Arenimonas*, *Silanimonas, Stenotrophomonas, Aquimonas*, *Panacagrimonas,* and *Pseudoxanthomonas* were characterized. In terms of *Arcobacter* assigned to *Campylobacteraceae* family, *Arcobacter cryaerophilus*, *Arcobacter trophiarum,* and *Arcobacter venerupis* were found. Moreover, higher abundance of *Enterobacteriaceae* was observed in Cave 2′s water sample in comparison with the rest of the samples where *Plesiomonas* (including *Plesiomonas shigelloides*), *Citrobacter* (including *Citrobacter murliniae*)*, Serratia, Providencia, Leclercia* (including *Leclercia adecarboxylata*)*,* and *Yersinia* were presented. More specifically, *Plesiomonas* (including *Plesiomonas shigelloides*) and *Serratia* were found in all samples. In addition, the presence of *Enterococcus*, *Cellvibrio* (including *Cellvibrio fibrivorans, Cellvibrio fulvus, Cellvibrio gandavensis, Cellvibrio japonicas, Cellvibrio mixtus,* and *Cellvibrio ostraviensis*) and *Pseudomonas* (<1%) was confirmed in Cave 2. In addition, unidentified *Enterococcaceae* and *Pseudomonadaceae* were detected (<1%) in Lake water and in Lake, Cave 1, and Cave 3.

Sequences assigned to pathogens or opportunistic pathogens were also detected in low abundance (<1%). In brief, *Legionellaceae* (<0.1%), *Coxiellaceae* (<0.1%), and *Vibrio* were detected in all samples, while the presence of *Legionella* (<0.1%) and *Mycobacterium* (<1%) was observed in Cave 3′s and Lake’s samples, respectively. In the case of the *Aeromonadaceae* family, which was found in samples collected from Lake and Cave 2, the identification at species level, i.e., *Aeromonas*, was succeeded only in Cave 2 samples.

## 4. Discussion

According to European and Greek legislation [10,12], the waters are not suitable for swimming prior to any disinfection, as all the tested parameters were above the required criteria. The only exception is that TVC grown on 37 °C were not significantly different from TVC grown on 22 °C. However, both counts were above the acceptable level (<200/mL) for thermal spring waters. Thus, it was clear from the microbial enumeration results that swimming should be prohibited in this area prior to any disinfection. Nevertheless, the characterization of the bacteria present in water could be useful to better understand the microbial ecology of the water.

In recent years, next-generation sequencing has been successfully applied to characterize the microbial communities of extremophiles and illustrate indicators of microbial contamination [14,15]. In this study, the metataxomomic analysis revealed a high microbial diversity in all samples. From a total of 166 OTUs detected, we detected sequences of important environmental, useful, and pathogenic bacteria or opportunist pathogens. However, several sequences failed to be identified at the genus or species level. It has to be noted that the presence of pathogenic bacteria should be confirmed in the future by quantitative PCR in order to further discuss the risk of swimming in contaminated waters more accurately. However, in this study, the potential impact of swimming in these waters on human health was investigated on the basis of the detection of bacterial sequences by metataxonomic analysis.

*Comamonadaceae*, *Sphingomonadadaceae*, and *Sinobacteraceae* are highly associated with biofilm formation, clean or chlorinated waters, and household water tanks [16,17,18,19,20]. From the detected genera of the *Comamonadaceae* family, the most common detected species, i.e., *Acidobacter*, is an important plant pathogen found in soil [21]. *Aquimonas voraii* and several species of the *Helicobacteraceae* family have been detected in waters of thermal springs, especially in environments with high sulfur and ferric concentrations [22,23]. Another commonly detected genus in the water environment is *Rhodobacter* [24], while its closest relative *Pseudorhodobacter* is also found in marine environments [25]. *Limnohabitans* and *Hydrogenophaga* are also highly associated with lake waters as they are an important part of bacterioplankton, soil, and water ecosystems [26,27,28]. The importance of *Hydrogenophaga* presence lies in the fact that strains of the genus were found to produce poly(3-hydroxybutyrate) or other copolymers and to contribute to environmental pollutant decomposition or removal [29,30,31]. Similarly, the main known denitrifiers, i.e., *Pseudomonas*, *Ralstonia*, *Alcaligenes*, *Rhodobacter*, *Rubrivivax*, *Thauera*, *Burkholderia*, *Bacillus*, *Hydromicrobium*, and *Streptomyces* [32,33] were also detected in this study. It was reported recently that species of *Cellvibrio* are able to degrade compounds such as cellulose, starch, chitin, and wood [34]. *Thiobacillus* and *Novosphingobium* species that can be detected in water environments are able to degrade sulfur and aromatic (phenol, nitrobenzene, etc.) compounds, respectively [35,36]. The ability of *Saprospiraceae* members to decompose complex organic compounds and their use for wastewater treatment has been reported previously [37]. Moreover, the composition of hyperchlorite from *Dechloromonas* could be an important factor for human health protection, considering that high hyperchlorite concentrations could lead to serious health issues including bone marrow disease [38].

Fecal-associated genera were also detected in this study. The water contamination could be associated with leakage of sewer pipes, domestic animals living in the area, or birds and wild animals [39,40]. It is well known that swimming in fecal contaminated waters can be associated with infections of the gastrointestinal tract, nasal system, ears, skin, and eyes; however, the infection dose is not well estimated [4]. *Escherichia coli* and *Enterococcus* are the indicators used to estimate fecal contamination. The presence of *Escherichia* in hot springs waters has been reported previously [41]. In this study, although *Enterococcus* were enumerated in all samples, meta-analysis of bacterial communities revealed that the *Enterococcocaceae* family was present only in waters collected from Lake and Cave 2. It has to be noted that the identification at species level was achieved in Cave 2′s waters, where higher numbers of *Enterococcus* were enumerated in comparison with the rest of the samples. This observation may depict the partial inability of next-generation sequencing techniques to detect species that are present in a low population in complex ecosystems. Nevertheless, the presence of *Enterococcus* in water is of importance, as the genus is associated with infections such as bacteremia, bacterial endocarditis, and meningitis [42]. Additionally, *Arcobacter* was the most commonly detected species in this study, being a potential risk factor for foodborne or waterborne human illness [43]. In several studies, *Arcobacter* was isolated from drinking, spring, lake water, and seawater [43,44,45,46,47,48,49]. Moreover, *Arcobacter cryaerophilus* and *Arcobacter butzleri* were isolated from patients with diarrhea [50,51] and spring water [43,52], respectively. In addition, farms and domestic animals are considered to be carriers of the important foodborne pathogen *Campylobacter* [53]. Thus, swimming waters may be contaminated due to waterflows from farms and/or residential areas; however, water chlorination could inhibit *Campylobacter*. In an earlier study, *Campylobacter* was detected in a geothermal spring [52]. Moreover, *Plesiomonas shigelloides,* the causing agent of gastroenteritis related to swimming in contaminated waters and seafood consumption [54], has been detected in this study. Similarly, the presence of *Citrobacter murliniae* was confirmed earlier in dead or sick animals’ ornithoids [55], while the opportunistic pathogenic genera *Serratia* and *Vibrio* were found to colonize the respiratory and urinary systems, wounds, and injuries, as well as being found to form biofilms [56,57,58]. The presence of *Serratia marcescens* in spring water has been reported previously [52]. The pathogenic species *Vibrio cholerae, Vibrio parahaemolyticus*, and *Vibrio vulnificus* are mainly transmitted from contaminated water [58]. The wound infection of an immunocompromised patient colonized by *Leclercia adecarboxylata* and *Enterobacter cloacae* has been associated, especially the former species, with swimming in contaminated waters [59].

One of the most important findings of this research is the detection of *Legionella* sequences. This is not the first time that *Legionella* has been observed in spring waters [41]. *Legionella*, along with *Pseudomonas aeruginosa*, are not associated with fecal infections. According to microbiological analysis, *Legionella* presumptive colonies were observed in all samples, while the presence of *Legionella* sequences was confirmed with metataxonomic analysis in three out of four sampling points. It is well known that *Legionella* spp. is the causing agent of legionellosis and other lung and non-lung diseases [4,60]. High temperatures and availability of nutrients found in spring waters create an ideal environment for the survival and growth of a high *Legionella* population [60,61]. In this study, *Pseudomonas* was also detected in three sampling points, while the *Pseudomonadaceae* family was detected in all cases but was not characterized at the species level in all samples. However, *Pseudomonas aeruginosa* sequences were not identified in this study. It is known that *Pseudomonas aeruginosa* could be found in water environments including spring waters, having been associated with infection caused by contamination through inhalation, wounds, eye, otitis, urinary system, etc. [4,62,63]. It has been reported previously that the human infection dose is close to 1000 CFU/mL [64,65], while it seems that the high temperature of spring waters facilitates the invasion of *Pseudomonas aeruginosa* in human body through the skin pores [66]. Moreover, genera belonging to *Coxiellaceae, Nocardioidaceae*, and *Clostridiaceae* families are considered to be potential human pathogens. It is of importance that the ultraviolet radiation during sunny days may reduce the *Clostridium* population [67]. On the other hand, extensive waterflows have been found to increase the population of *Clostridium perfringens*, coliforms, *Escherichia coli*, and *Enterococcus* [68]. Another important human pathogenic genus that was detected is *Aeromonas*. The washing of wounds with contaminated water may lead to limb amputation if an antibiotic treatment is not applied [69]. Similarly, the detection of putative pathogenic bacterium *Mycobacterium* has to be taken into consideration as it causes skin infections and is able to easily multiply in water environments [6,70]. *Mycobacterium* was also detected in hot springs [41], while the association of pneumonia with *Mycobacterium avium* after sauna use has been reported previously [71]. High resistance of *Mycobacterium avium* to disinfectants [72] may pose as a weakness of ineffective disinfection strategies. The availability of different treatments and potential strategies to disinfect spas and related environment has been well reviewed recently [7].

## 5. Conclusions

Microbial enumeration exhibited that the spring waters in question were contaminated, and thus the decision to prohibit swimming prior to any disinfection in the area is accurate. In general, metataxonomic analyses were in agreement with microbial enumeration of the targeted population, although in some cases, bacteria present in low population were not detected by next-generation sequencing. Nevertheless, the deep characterization of microbial communities gave a better insight into the bacteria present in thermal spring waters. It is of importance that sequences of other pathogenic bacteria or opportunistic pathogens that were not measured according to legislation were detected. However, the presence of pathogenic bacteria should be confirmed with quantitative PCR in the future. Through this information, the disinfection strategy, which aims to be achieved through the modernization of the existing facilities and infrastructure networks of the area, could be improved. Targeting the most resistant microorganisms could be a proper management of the applied disinfection method and of rehabilitation actions in general.

## Figures and Tables

**Table 1:** Population of microbiological parameters enumerated in thermal waters of Lake, Cave 1, Cave 2, and Cave 3 of Kaiafas thermal spring Anigrides Nymphes in Greece.

Microbiological Parameter	Population (CFU/100mL Water)				
	Sampling Point				Thermal Water Parameter’s Value [10,12]
	Lake	Cave 1	Cave 2	Cave 3	
	Mean Value ^1^ [Lowest–Highest] ^2^	Mean Value [Lowest–Highest]	Mean Value [Lowest–Highest]	Mean Value [Lowest–Highest]	
Total viable counts (22 °C)	253,333 [170,000–440,000]	185,000 [140,000–230,000]	5,630,000 [1,490,000–11,000,000]	1,000,000 [420,000–2,100,000]	<200
Total viable counts (37 °C)	360,000 [160,000–640,000]	250,000 [160,000–340,000]	5,810,000 [830,000–12,100,000]	916,667 [360,000–1,980,000]	<200
*Escherichia coli*	95 [75–106]	71 [66–75]	495 [376–720]	300 [142–536]	0
Coliforms	10,833 [9400–11,600]	6050 [5400–6700]	274,667 [28,000–760,000]	42,900 [11,700–95,000]	<15
*Enterococcus*	3000 [1000–7000]	2044 [1088–3000]	45,333 [7000–113,000]	26,000 [5000–40,000]	<0 (value for drinking water)
*Pseudomonas aeruginosa*	108 [60–170]	61 [51–71]	153 [99–195]	122 [116–132]	<10
*Legionella* (presumptive colonies) ^3^	7069 [5689–8448]	3368 [2037–4704]	3532 [2224–4840]	3200 [2384–4016]	<1

^1^ Mean value of triplicates. ^2^ Lowest and highest enumerated population. ^3^ Colonies enumerated on Buffered Charcoal Yeast Extract (BCYE) *Legionella* agar.

## Supplementary Materials

The following are available online at https://www.mdpi.com/1660-4601/17/23/9133/s1, Figure S1: Map of Kaiafas thermal spring Anigrides Nymphes located in regional unit of Ileia of Western Greece Prefecture (Greece). The sampling points (Lake, Cave 1-3) are indicated by the yellow pins (left figure). The sampling points Cave 1-3 are indicated on the topography plan of Anigrides Nymphes [Spinos, J.; Agouridis, C.; Casati, L.; Dell Oro, B.; Bolanz, J.; Giannopoulos, V.; Deriaz, P. 1989] (right figure), Figure S2: Principal component analysis (PCA) 3D score plot based on bacterial families detected in thermal waters of Lake, Cave 1, Cave 2, Cave 3 of the Kaiafas thermal spring Anigrides Nymphes in Greece by metataxonomic analysis. The explained variances are shown in brackets. The total variance of the data set (100%) could be explained by the 3 first PCs.
