# Supplementary material for: Exploring the Bacterial Communities of the Kaiafas Thermal Spring Anigrides Nymphes in Greece Prior to Rehabilitation Actions
Source: Int J Environ Res Public Health. 2020 Dec 7;17(23):9133. doi: 10.3390/ijerph17239133 (PMC7730472; doi:10.3390/ijerph17239133)
Supplement: Supplementary file 1 [file ijerph-17-09133-s001.pdf]

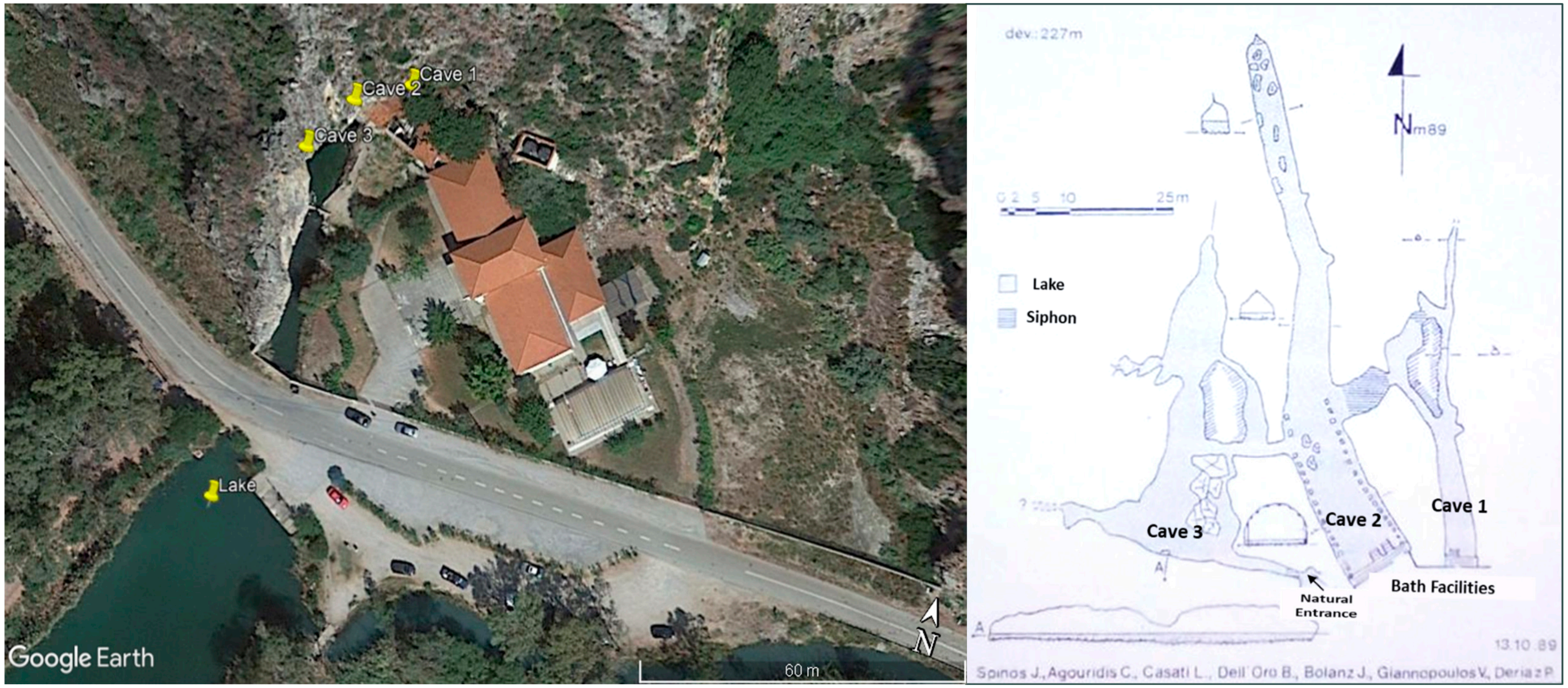

Supplementay Figure 1. Map of Kaiafas thermal spring Anigrides Nymphes located in regional unit of Ileia of Western Greece Prefecture (Greece). The sampling points (Lake, Cave 1-3) are indicated by the yellow pins (left figure). The sampling points Cave 1-3 are indicated on the topography plan of Anigrides Nymphes [Spinos, J.; Agouridis, C.; Casati, L.; Dell Oro, B.; Bolanz, J.; Giannopoulos, V.; Deriaz, P. 1989] (right figure).
